# Influence of full mouth rehabilitation on oral health‐related quality of life among disabled children

**DOI:** 10.1002/cre2.78

**Published:** 2017-09-08

**Authors:** Abeer M. Al‐Nowaiser, Abdulaziz S. Al Suwyed, Khalid H. Al Zoman, Asirvatham A. Robert, Tarfa Al Brahim, Sebastian G. Ciancio, Sultan A. Al Mubarak, Omar A. El Meligy

**Affiliations:** ^1^ Pediatric Dentistry Department King Abdulaziz University Saudi Arabia; ^2^ Dental Department King Abdulaziz Medical City Saudi Arabia; ^3^ Dental Department King Faisal Specialist Hospital & Research Center Saudi Arabia; ^4^ Department of Endocrinology and Diabetes Diabetes Treatment Center, Prince Sultan Military Medical City Saudi Arabia; ^5^ Department of Nutrition Princess Nourah bint Abdulrahman University Saudi Arabia; ^6^ Department of Periodontics and Endodontics, School of Dental Medicine State University of New York at Buffalo New York USA; ^7^ Pediatric Dentistry and Dental Public Health Department Alexandria University Egypt

**Keywords:** disabled children, follow‐up, full mouth rehabilitation, oral health, oral hygiene, quality of life

## Abstract

The efficacy of full mouth rehabilitation (FMR) on oral health‐related quality of life of physically disabled children was assessed. This prospective study was performed at Dental Department of Sultan Bin Abdulaziz Humanitarian City, Riyadh, and King Abdulaziz University Hospital, Jeddah, Saudi Arabia, during May 2012 to September 2014. A total of 186 physically disabled children aged 11–14 years were assigned to a test group (n = 97) or a control group (n = 89). FMR was applied for test group children at baseline and 3 months' visits, whereas those in the control group did not receive FMR. Both group children received dental kits and oral hygiene instructions. Children were asked to complete the Child Perceptions Questionnaire, whereas Parental‐Caregiver Perceptions Questionnaire and Family Distress Domain questionnaire were completed by the parents/caregivers at baseline and 6 months' visits. Children in both groups showed positive trends in oral symptoms at 6 months compared with those at baseline. However, when they were compared to control, significant improvement in oral symptoms was observed in the test group at 6 months' visit (p < .05). Also when they were compared to control, significant improvements were observed in the functional limitation, emotional, and social well‐being subscales of the Child Perceptions Questionnaire and on the Parental‐Caregiver Perceptions Questionnaire scales at the end of the study (p < .05). Compared to the parents/caregivers of the control children, the parents/caregivers of the test‐group children reported insignificant but positive trends in Family Distress Domain at the end of the study (p < .05). FMR in children reduced oral‐related problems subsequently to a better oral health‐related quality of life.

## 1 INTRODUCTION

The past few decades have exposed the fact that oral health‐related quality of life (OHRQoL) is significantly impacted by clinical dentistry and dental research (El Ashiry, Alaki, & Nouri, 2016; Birungi et al., 2016; Reissmann, John, Sagheri, & Sierwald, 2016). The OHRQoL is a multidimensional concept involving the subjective assessment of the individual's oral health, functional and emotional well‐being, anticipation and satisfaction with care, and the understanding of self (Adeniyi, Diaku‐Akinwumi, & Ola, 2016; Alsumait et al., 2015). Measuring a child's OHRQoL facilitates the assessment of the child's oral health status and treatment efficacy. Prior studies revealed that good oral health, implying disease‐free and intact the teeth, gums, and oral mucosal tissues, is an important aspect of overall health, particularly significant for children with special health needs (Norwood & Slayton, 2013).

It is well demonstrated that oral hygiene causes problems with aesthetics and communication, with solid biological, psychological, and social implications (Emami, de Souza, Kabawat, & Feine, 2013). Sadly, oral health care ranks among the most ignored health needs of disabled individuals (Bartolome‐Villar, Mourelle‐Martinez, Dieguez‐Perez, & de Nova‐Garcia, 2016; Dieguez‐Perez, de Nova‐Garcia, Mourelle‐Martinez, & Bartolome‐Villar, 2016; Roberts, Chetty, Kimmie‐Dhansay, Fieggen, & Stephen, 2016). Oral health of the disabled could be overlooked due to either the disabilities themselves or more likely to their restricted access to oral health care (Dieguez‐Perez, et al., 2016; Roberts, et al., 2016; Jaber, Sayyab, & Abu Fanas, 2011). Moreover, due to their limited ability to handle oral examinations, disabled individuals encounter distinct challenges in obtaining oral health care services (Roberts et al., 2016). However, with correct forethought, clear communication, and accurate assessment of the restrictions to the services provided, the considerable disregard for dental care in the majority of these individuals can be mitigated (Ivancic Jokic, Majstorovic, Bakarcic, Katalinic, & Szirovicza, 2007; Jaber, et al., 2011).

Disabled children, including those with states of health that influence their behavior and cognition, are most often limited in their capacity to accomplish their normal activities of daily living. They may also have special health care needs (Krause, Vainio, Zwetchkenbaum, & Inglehart, 2010; Moursi, Fernandez, Daronch, Zee, & Jones, 2010). However, dentists face tremendous challenges when they are required to treat young disabled children with severe dental problems, particularly when extensive and complicated treatment is needed (Jokovic et al., 2002). Pediatric dentists who have undergone specific training to treat disabled individuals are very few in number and unequally distributed across Saudi Arabia to be sufficient to satisfactorily deal with the treatment requirements of these patients. Therefore, general dentists lacking such specialized training have no choice other than to treat such children with special health care needs in their practices.

Although several studies on the oral health of the disabled population in Saudi Arabia have been performed, a limited number of studies have focused particularly on the influence of dental hygiene on the quality of life in this specific group. The objective of this study, therefore, was to assess the efficacy of full mouth rehabilitation (FMR) on OHRQoL of physically challenged children populace in Saudi Arabia.

## 2 METHODS

This prospective study was performed at the Dental Department of Sultan Bin Abdulaziz Humanitarian City, Riyadh, and King Abdulaziz University Hospital, Jeddah, during May 2012 to September 2014, both of which are tertiary care centers that cater to the disabled population. For the purpose of this study, children were considered to have a physical disability if they exhibited a substantial limitation in the ability to perform basic physical activities, such as walking, climbing stairs, reaching, lifting, or carrying (Disability Statistics: Online Resource for US Disability Statistics, the Employment and Disability Institute, 2000).

Children were considered eligible for inclusion if they had a physical disability and a minimum of 12 primary and/or permanent teeth that had not been treated over the previous 12 months. Children who were participating in any other concurrent clinical trials were not eligible for this study, and those with serious medical conditions or with transmittable or malignant diseases were also not eligible. The children or their parents/caregivers were advised of their roles in this study and were required to sign an informed consent form prior to being recruited into the study. The informed consent included a pediatric evaluation prior to any interventional treatment. However, children with dental disease that required immediate intervention (i.e., pain and swelling) were not considered for this study and were treated as appropriate within the facility.

To obtain good intraexaminer and interexaminer reliability, the examiners were calibrated prior to baseline registration. Ten children were examined, then they were reexamined a week later, and the level of agreement between corresponding readings was assessed using the kappa method.

A total of 214 physically disabled children aged 11–14 years were consecutively recruited into the study, and each child was followed up for a period of 6 months from initial recruitment. The children at both hospitals were allocated numbers, which were used to randomize the different groups. This was achieved by distributing the patient number among the test and control by sequentially allocating them to one of two alphabetical codes relating to a test group and a control group (i.e., A for test group and B for control group). Of these 214 children, 116 were in the test group and 98 were in the control group. A total of 28 children (19 in the test group and 9 in the control group) were ultimately excluded from the study, for reasons that violated the inclusion criteria—that is, taking an antibiotic during the study period, emergency extraction of tooth/teeth, minor or major surgical intervention during the study period, or due to loss of contact. Children in the test group received direct oral health care treatment (as defined below), whereas those in the control group did not receive any such treatment during the study period, except for emergency situations. OHRQoL measurements were assessed at the baseline visit (0 months) and at the 6‐month visit.

The height and weight of each child were measured, and their body mass index (BMI; kg/m^2^; Table 1) and BMI *z* score (BMI adjusted for age and sex) calculated. The BMI *z* score (or standard deviation [SD] score) was calculated using the formula (Xi Mx)/SD, where Xi is the BMI, Mx is the mean BMI value for the age and sex of the child, and SD is the standard deviation corresponding to that age and sex. Childrenname, gender, age, address, and contact information were recorded, to enable us to contact these patients for future research after 2 years, to evaluate any long‐term changes in their periodontal status. These data will become part of a reliable database regarding long‐term progression of the disease in the Saudi population.

**Table 1:** Demographic data

Variables	Test group (*n* = 97)	Control group (*n* = 89)
	*n* (%)	*n* (%)
Gender		
Male	57 (58.8)	52 (58.4)
Female	40 (41.2)	37 (41.6)
Age (years)		
11	33 (34.0)	26 (29.2)
12	28 (28.9)	25 (28.1)
13	22 (22.7)	23 (25.8)
14	14 (14.4)	15 (16.9)
BMI		
Underweight	12 (12.4)	9 (10.1)
Normal	65 (67.0)	62 (69.7)
Overweight	15 (15.5)	13 (14.6)
Obese	5 (5.1)	5 (5.6)

*Note*. BMI = body mass index.

### 2.1 Dental treatment

After screening and examination visits, FMR was applied for test group children at baseline and 3 months' visits. It should be considered here that baseline visits consist of one to three separate visits within maximum of 7 days' period. The baseline visits for the test group involved combination of the following several treatment modalities: 1, conservative adhesive restorations; 2, restorations of primary teeth/or permanent teeth; 3, fissure sealant; 4, root canal treatment (pulpotomy and pulpectomy); 5, stainless steel crowns; 6, extraction of nonrestorable teeth; and 7, prosthetic dental treatment, including partial dentures, space maintainers, and space regainers. All children received dental kits containing an electric toothbrush, toothpaste, tongue cleaners, and mouthwash. Oral hygiene instructions were given to all children during all the follow‐up visits. Each child's medical and dental history was considered to determine how their specific dental rehabilitation would be performed using the aforementioned techniques.

Full mouth rehabilitation was performed by qualified pediatric dentist residents supervised by their consultants. Dental treatment was done for all affected teeth, in addition to preventive treatment for sound teeth, under local anesthesia in the dental clinic for cooperative children, under conscious sedation in the dental clinic for moderately uncooperative children, or under general anesthesia for extremely uncooperative children who could not be treated in the dental clinic. Efforts were undertaken to finish the treatments at a single visit for uncooperative children who were treated under conscious sedation and/or under general anesthesia at the operating room and in as few visits as possible for all other children. It should be considered here that baseline visits consist of one to three separate visits within maximum of 7 days' period (32 children completed treatment in one visit, 47 children in two visits, and 37 children in three visits).

### 2.2 Oral hygiene evaluation

A personal oral hygiene evaluation checklist was used to evaluate the disabled child's ability to maintain his or her oral care. The person who worked most closely with the child completed this form, which was subsequently reviewed by the dentist or hygienist to solicit recommendations for oral hygiene and care.

Oral hygiene status was recorded using the special plaque index by visually evaluating the presence of plaque on the buccal and lingual surfaces of upper and lower incisors and canines. Teeth were classified as “good” if no plaque was visible, “fair” if there was a small quantity of plaque or recent food accumulation, and “poor” if there was considerable plaque or long‐standing accumulation of food (James, Iaekson, Slack, & Lawton, 1960). Oral hygiene status was recorded at baseline and at 3‐ and 6‐month postoperative visits.

### 2.3 Quality of life

We developed a questionnaire, which was completed by asking the parents/caregivers, to determine the frequency of various oral health‐related impacts on QoL for children with special health care needs aged 5–14 years. It was a modification of the Child Perceptions Questionnaire for children aged 11 to 14 years (CPQ11–14) that was originally developed and validated in Toronto, Canada (Jokovic et al., 2002). This modification was based upon compatibility with the Saudi population as well as the disabilities associated with the target group. It was professionally translated into Arabic, revised twice, and translated back into English for verification. The validity and reliability of the modified Arabic translation of the CPQ11–14 used in this study were assessed preoperatively. Two general classes of reliability were assessed for this study: (a) interrater reliability (interexaminer) assesses the degree of agreement between two or more raters in their appraisals. Interexaminer reliability was determined using the kappa method and was found to be 0.98, which represents excellent agreement. (b) Test–retest reliability (intraexaminer) assesses the degree to which test scores are consistent from one test administration to the next. Measurements are gathered from a single rater who uses the same methods or instruments and the same testing conditions. Intraexaminer reliability was also determined and was 0.98, representing excellent agreement. Regarding validity, construct validity was assessed, which refers to the extent to which operationalizations of a construct (i.e., practical tests developed from a theory) do actually measure what the theory says they do. As an index of construct validity, Pearson's correlation was highly significant at the 0.01 level. The questionnaire was delivered to the parents/caregivers at baseline and at the 6‐month postoperative follow‐up visit. It contained a battery of 24 questions divided into four health domains: oral symptoms (*n* = 7), functional limitations (*n* = 7), emotional well‐being (*n* = 3), and family well‐being/parental distress (*n* = 7).

Each question asked about the frequency of these events over the previous 3 months in relation to the child's oral/oro‐facial condition. The response options were assigned numbers from 0 to 4: *never* = 0; *once/twice* = 1; *sometimes* = 2; *often* = 3; *every day/almost every day* = 4. The answers were scored and linearly transformed to a 0–100 scale (0 = *0*, 1 = *25*, 2 = *50*, 3 = *75*, and 4 = *100*). The total score on the scale is directly proportional to the impact of oral status on the adolescent's quality of life (Jokovic, Locker, & Guyatt, 2006).

### 2.4 Family distress

The parents/caregivers of children independently completed the Family Distress Domain (FDD) questionnaire, which included the following types of questions: Have you been upset? Has your sleep been disrupted? Have you felt guilty? Have you taken time off from work (e.g., for pain, appointments, and surgery)? Have you had less time for yourself or the family? Are you worried that your child will have fewer life opportunities? Have you felt uncomfortable in public places (e.g., stores and restaurants) with your child? The FDD questionnaire was completed at both the baseline and 6‐month visits, and any changes in FDD scores were assessed between the treatment groups (Li, Malkinson, Veronneau, & Allison, 2008).

### 2.5 Withdrawals and dropout

Participants were free to withdraw from the study at any time if they so wished. Participants were registered as a dropout if they were absent from or unable to keep an appointment as planned. The reasons for each withdrawal/dropout were stated on their form.

### 2.6 Statistical analysis

A bio‐statistician was consulted during the planning stages and after collecting data for analysis. Data analysis was carried out using Microsoft Excel 2010 (Microsoft Corporation, Seattle, WA, USA) and Statistical Package for Social Sciences version 16 (SPSS Inc., Chicago, IL, USA). Data were organized and presented numerically, graphically, and mathematically. Various statistical methods were used; that is, the Kolmogorov–Smirnov test was performed for equal variances across the groups, and *t* test and paired *t* test were conducted to look at differences carried out for making comparisons among the groups. The level of significance was set at 0.05 with a 95% confidence interval (a *p* value of less than 0.05 was considered statistically significant).

### 2.7 Ethical considerations

This study has been approved by the ethics committee of the Sultan Bin Abdulaziz Humanitarian City, Riyadh, Saudi Arabia, and has therefore been performed in accordance with the ethical standards laid down in the 1964 Declaration of Helsinki and its later amendments (project number 023).

## 3 RESULTS

Calibration results by the examiners were found to be in excellent agreement (*k* = 0.98). As an index of construct validity, Pearson's correlation was highly significant at the 0.01 level for total scale and oral symptoms (*r* = 0.71), functional limitations (*r* = 0.86), emotional well‐being (*r* = 0.73), and family well‐being (*r* = 0.81).

### 3.1 Study population

A total of 186 (97 in the test group and 89 in the control group) disabled children were included in the study (Table 1). The BMI of the majority of the study population was within a healthy range (Table 1).

The oral hygiene skills of the study population are shown in Table 2. As determined from the completed oral hygiene checklists, overall, 41% of children required significant assistance when cleaning their teeth, with 28% brushing effectively, with little assistance. With respect to the toothbrush type used by the children, 61% used manual toothbrushes, whereas 23% used electric brushes. Almost 67% of children used toothpaste appropriately, and 48% were able to floss.

**Table 2:** Oral hygiene skills among the test group

Variables	Skill	%
Classification of cleaning skills	Requires significant assistance	41
	Has some dexterity but uses insufficient cleaning techniques	16
	Effectively brushes with little assistance	28
	Requires virtually no assistance	15
Current brushing method	Manual toothbrush	61
	Electric	23
	Specially designed toothbrush	9
	Cleans dentures properly	7
Uses toothpaste appropriately	Yes	67
	No	33
Rinsing	Rinses toothpaste from mouth/uses mouthwash	36
	Unable to rinse	64
Flossing	Able to floss	48
	Unable to floss	52

Tooth loss and body weights for children in both study groups are shown in Table 3. Compared to children with no tooth loss, significant differences were observed in children with ≥6 teeth regarding the mean body weight of test group (*p* < .05). Similar results were also observed in the control group (*p* < .05).

**Table 3:** Tooth loss and body weight of the study population

Tooth loss	Test group weight (kg, mean ± SD) *n* = 97			Control group weight (kg, mean ± SD) *n* = 89		
	Baseline	6 months	*p* value	Baseline	6 months	*p* value
No loss	19.3 ± 2.3	21.7 ± 2.5	<.0001*	17.9 ± 1.67	19.8 ± 1.45	<.0001*
1 tooth	18.7 ± 2.5	21.3 ± 2.4	<.0001*	17.1 ± 1.72	20.5 ± 1.63	<.0001*
2–3 teeth	19.1 ± 1.97	21.6 ± 2.1	<.0001*	18.3 ± 1.97	21.4 ± 2.32	<.0001*
4–5 teeth	21.8 ± 2.3	23.2 ± 1.8	<.0001*	20.7 ± 2.23	23.8 ± 2.21	<.0001*
≥6 teeth	24.8 ± 2.1	26.1 ± 1.7	<.0001*	23.9 ± 2.16	26.8 ± 2.43	<.0001*

*Note*.

* Statistically significant at *p* < .05.

**Figure 1:**
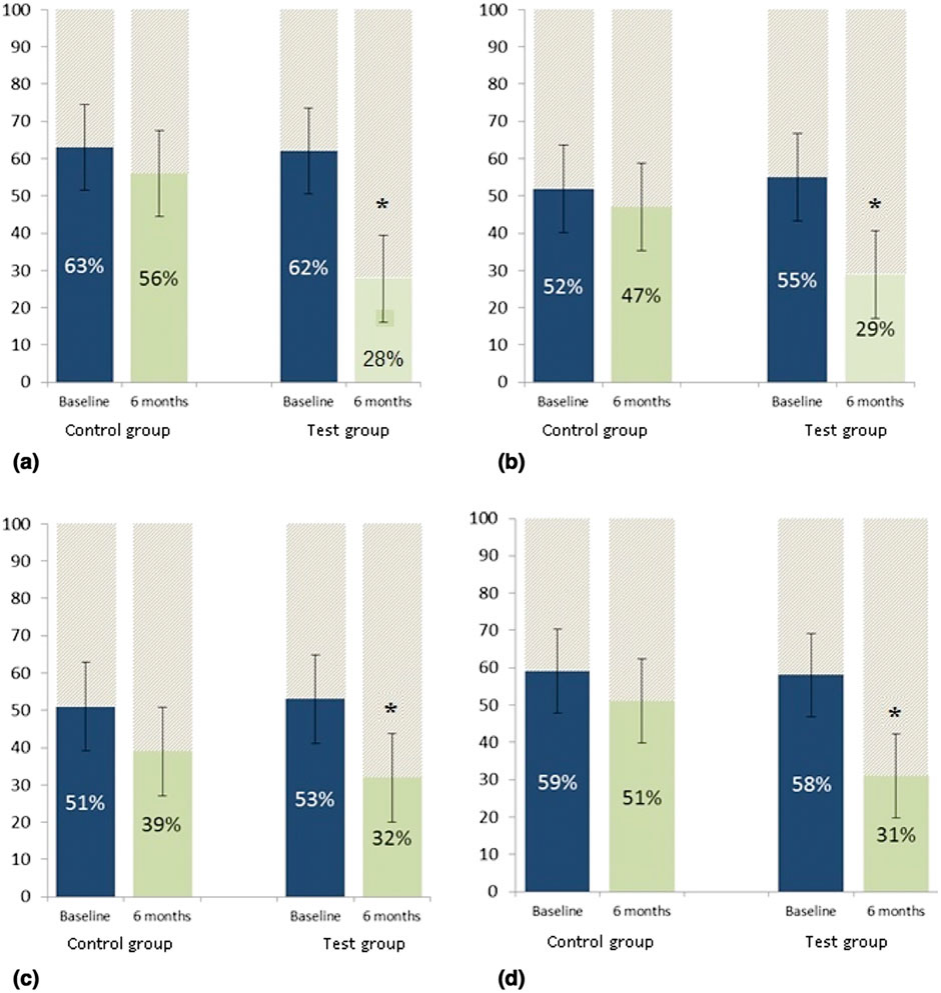
Measures of oral health‐related quality of life among children aged 11–14 years: (a) oral symptoms, (b) functional limitations, (c) emotional well‐being, and (d) social well‐being

The results from CPQ11–14 showed that children in both study groups experienced improvements in their oral symptoms at 6 months in comparison to baseline (Figure 1a), and in the case of the test group, a significant improvement was observed at 6 months (*p* < .05). A significant improvement was also observed in the functional limitation of the test group children at 6 months compared to baseline (29% vs. 56%; *p* < .05; Figure 1b). Similar positive trends were also found on the emotional and social subscales of the CPQ11–14, particularly for children in the test group (Figure 1c,d).

**Figure 2:**
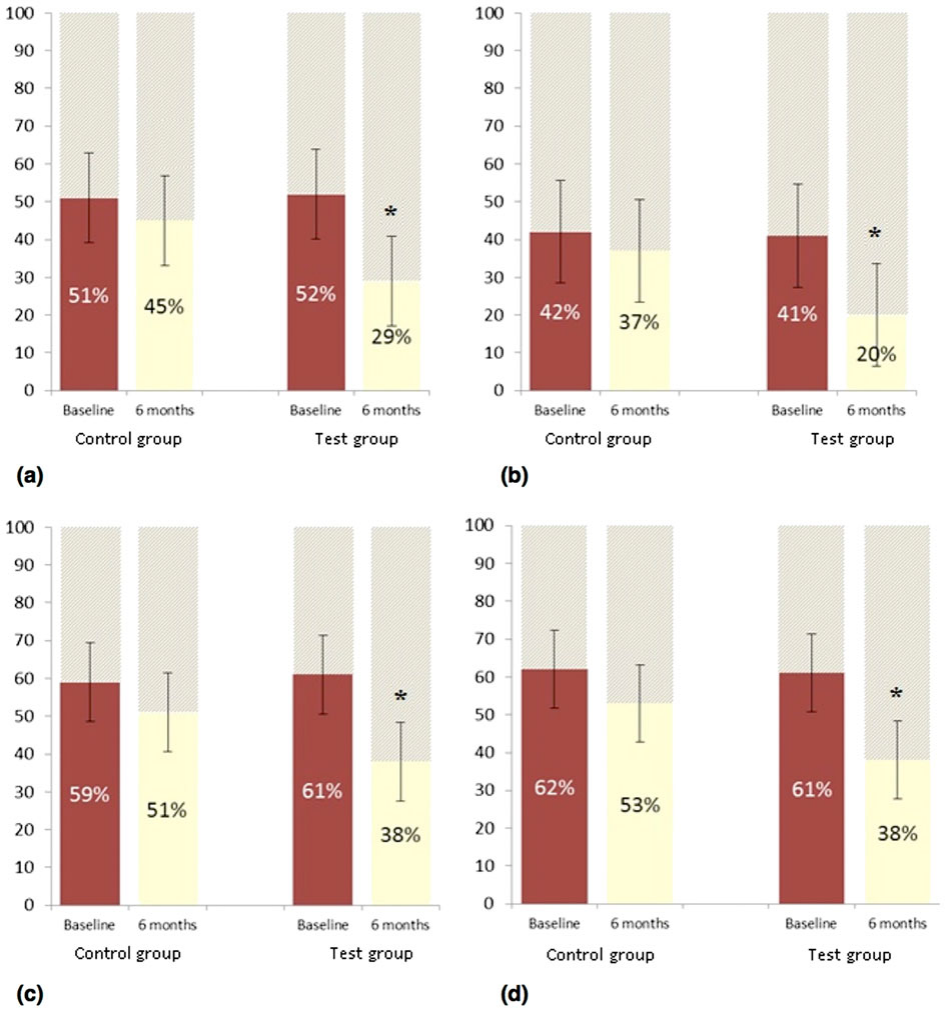
Measures of parental perceptions of child‐related quality of life: (a) oral symptoms, (b) functional limitations, (c) emotional well‐being, and (d) social well‐being

The results from the Parental‐Caregiver Perceptions Questionnaire (P‐CPQ), which was completed by the children's parents/caregivers, showed similar trends to those described for the CPQ11–14 (Figure 2a–d). No significant differences were found between the CPQ11–14 and P‐CPQ scores.

**Figure 3:**
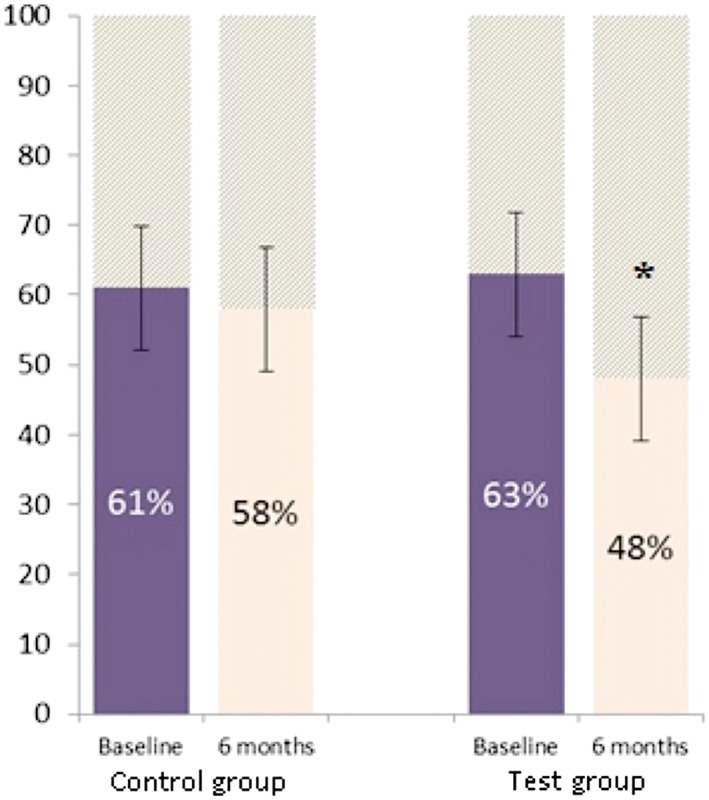
Family Distress Domain

No significant differences were found in FDD scores between the two study groups at baseline (Figure 3). However, at the end of study, insignificant but positive differences in FDD scores were observed from parents/caregivers of the test group children compared to the parents/caregivers of control the children.

## 4 DISCUSSION

There are many reports available in the dental literature on oral health surveys done in normal children, which is not a new research field; however, there are relatively limited data on the oral conditions in physically challenged children and adolescents. Therefore, this research assessed the extent of the FMR on OHRQoL of physically disabled children in Saudi Arabia.

Research reveals that poor oral health in disabled children has an adverse effect on their nutrition, digestion, ability to chew and relish food, facial shape, and speech, with subsequent negative effects on QoL. Further, studies on the prevalence of oral disease in disabled children groups showed substantially low oral hygiene levels in the disabled population (Al‐Maweri & Zimmer, 2015; Gace, Kelmendi, & Fusha, 2014; Gardens et al., 2014), a fact that this study confirmed, in which poor oral symptoms were recorded in disabled children. In their review of 32 studies on disabled children, Brown and Schodel (2014) reported that disabled children are prone to have poorer oral hygiene than are their nondisabled fellows. In a recent study, Al‐Maweri and Zimmer (2015) reported that children with disabilities show high propensity for dental caries and low oral hygiene. Physically disabled children showed the highest rate of dental caries in comparison with visual and hearing impaired children. Children normally exhibit a wide repertoire of brushing ability, associated with coordinated muscular movements, inherent skills, the ability to comprehend and process instructions, and age (Unkel, Fenton, Hobbs, & Frere, 1995). Physically disabled children can find it difficult to use a manual toothbrush (Shaw, Maclaurin, & Foster, 1986). Obviously, the standard of oral hygiene declines with the intensity of intellectual disability, which appears to substantiate a relationship between the degree of oral hygiene and the intensity of the disability. The present study revealed poor oral hygiene skills (i.e., rinsing, flossing, and using toothpaste appropriately) among disabled children. However, in a study, dental health behavior, the utilization of floss and toothpicks, and oral rinsing were found to be unrelated in multivariate analyses on problems related to the oral quality of life (Dahl, Wang, Skau, & Ohrn, 2011). This present study found it remarkable that children with fewer numbers of teeth showed higher body weight. One hypothesis proposed as an explanation for this observation is that sweetened drinks, which could lead to teeth loss, are high in energy (kilojoules) and could, therefore, cause the excessive weight gain in children, if regularly consumed in large quantities. Also, physical deficits often prevent disabled children from participating in particular kinds of sports or recreational sports with healthy children and adolescents. Poor physical fitness further impede active participation in sports groups. A lack of available facilities for disabled people constitutes another factor that precludes active exercise. In view of the many barriers to exercise, it is hardly surprising that disabled children consume more television and computer games (Rimmer, Rowland, & Yamaki, 2007).

In the present study, children in both groups (test and control) revealed positive trends in the oral symptoms and functional ability at 6 months of age when compared with the baseline. However, only the group that received full oral rehabilitation registered marked improvement. Similar positive trends were also seen in the emotional and social well‐being CPQ11–14 subscales. This study showed similar results for the P‐CPQ, with no significant difference being observed between the CPQ11–14 and P‐CPQ values. Jankauskiene and Narbutaite (2010) concluded that dental treatment enhances the children's QoL in the physical, psychological, and social aspects. They also reported that dental treatment affects the entire family of the patient positively, with appreciation by the parents, similar to the findings in this study. Scores on the child's perception of oral symptoms, functional limitations, emotional well‐being, and social well‐being (i.e., OHRQoL scores) were reportedly worse than the parents' perceptions of their child's OHRQoL scores. However, such differences were inconsequential. Jokovic, Locker, Stephens, and Guyatt (2003) in a study of 42 mothers and their children reported good concurrence between the groups. However, appreciable inconsistencies between pairs were also noted, particularly with respect to both the emotional and social well‐being, implying that mothers cannot act as proxies for their children at an individual level. In the functional realm, mothers misjudged the level of hardship faced by their children in eating and the duration of that task, as well as the rate of mouth breathing compared to their children. Additionally, Jokovic et al. and Benson, O'Brien, and Marshman (2010) observed low concurrence between the children and their mothers with respect to this query on the functional domain. Malden, Thomson, Jokovic, and Locker (2008) in a pilot study showed that disabled children showed good headway posttreatment under general anesthesia for all the OHRQoL indicators. Parents reported good improvement in their children's quality of life connected with eating and sleeping, postdental intervention.

Reports of elevated levels of depressive symptoms and feelings of amplified psychological distress were observed in the mothers of children with chronic illnesses or disabilities (Emerson & Llewellyn, 2008). A research done on the parents of disabled children revealed clinical signs of depression in a majority of the mothers on assessment (Laxman et al., 2015). The present study also confirmed that higher levels of parental distress were recorded in the parents/caregivers of disabled children. Although no significant differences were found in the FDD at baseline after 6 months, the parents/caregivers of the children in the test group showed higher but significant FDD scores than did the control group parents/caregivers, thus supporting both the children and their parents/caregivers. The major limitations of this study include the following: limited number of risk factors examined and performed at two centers. More studies on a larger scale are needed to address the limitations indicated in the study.

## 5 CONCLUSIONS

Oral rehabilitation in disabled children was found to enhance their oral health as well as the physical, emotional, and social quality of life. Also, children who suffered no tooth loss showed significant differences from those who experienced tooth loss, having ≥6 teeth, with respect to mean body weight.

## RECOMMENDATIONS

Further research is necessary to measure the long‐term OHRQoL changes, as well as study and survey such children.
